# Long-term radiographic outcomes and functional evaluation of ulnar shortening osteotomy in patients with ulnar impaction syndrome and reverse oblique sigmoid notch: a retrospective case series study

**DOI:** 10.1186/s12891-021-04029-7

**Published:** 2021-02-03

**Authors:** Hui-Kuang Huang, Steve K. Lee, Yi-Chao Huang, Cheng-Yu Yin, Ming-Chau Chang, Jung-Pan Wang

**Affiliations:** 1 Department of Surgery, School of Medicine, National Yang-Ming University, Taipei, Taiwan; 2 Department of Orthopedics & Traumatology, Taipei Veterans General Hospital, Taipei, Taiwan; 3 Department of Orthopedic Surgery, Ditmanson Medical Foundation Chiayi Christian Hospital, Chiayi, Taiwan; 4 Chung Hwa University of Medical Technology, Tainan, Taiwan; 5 Department of Orthopedic Surgery, Hand and Upper Extremity Service, Hospital for Special Surgery, New York, NY USA; 6 Department of Orthopedic Surgery, National Yang-Ming University Hospital, Yilan, Taiwan

**Keywords:** Ulnar impaction, Oblique, Reverse, Sigmoid notch, Ulnar shortening

## Abstract

**Background:**

Ulnar shortening osteotomy (USO) is an effective treatment for ulnar impaction syndrome. However, there have been reports of osteoarthritis (OA) at the distal radioulnar joint (DRUJ) when USO was performed on patients with a reverse oblique sigmoid notch. This study aimed to evaluate the radiographic and functional outcomes following USO in patients with a reverse oblique sigmoid notch.

**Methods:**

We retrospectively reviewed patients having a reverse oblique sigmoid notch who underwent USO for ulnar impaction syndrome between 2002 and 2013. We evaluated radiographic changes of the DRUJ and functional outcomes of patients.

**Results:**

We enrolled 22 patients (22 wrists) with an average age of 49.6 years and a mean follow-up of 93.2 (range, 36–179; standard deviation [SD], 38.2) months. We found that there were changes in the inclination angle of the sigmoid notch, from an average reverse oblique of 14.9^o^ (range, 11^o^–23^o^; SD, 3.4^o^) preoperatively to a more parallel 5.1^o^ (range, 0^o^–11^o^; SD, 3.2^o^) at the final follow-up. The functional results at the final follow-up were good, with a mean visual analogue scale for pain of 0.2 (range, 0–1; SD, 0.4) at rest and 1.3 (range, 0–3; SD, 0.9) during activity, QuickDASH of 15.1 (range, 2.3–34.1; SD, 8.8), and modified Mayo Wrist Score of 91.6 (range, 70–100; SD, 6.4). Seven wrists (31.8%) had changes compatible with OA, but the wrists did not exhibit a significantly worse function when compared to wrists without OA changes, except for supination motion and grip strength.

**Conclusions:**

For patients with a reverse oblique sigmoid inclination following USO, we observed that the inclination angle had a tendency to become parallel and some patients developed OA at the DRUJ. However, long-term functional outcomes could still be good. The reverse oblique sigmoid inclination does not seem to be an absolute contraindication for USO.

**Supplementary Information:**

The online version contains supplementary material available at 10.1186/s12891-021-04029-7.

## Background

Ulnar shortening osteotomy (USO) is a commonly performed procedure that can treat many ulnar-sided wrist problems, including ulnar impaction syndrome, triangular fibrocartilage complex (TFCC) problems, instability of the distal radioulnar joint (DRUJ), and lunotriquetral ligament tear [1–5]. According to Scheker and Severo, USO can also be used in early osteoarthritis (OA) to slow disease progression and reduce the need for prosthetic replacement or salvage procedures [6]. USO was found to unload the ulnar-sided wrist from the carpus and tighten the ulnocarpal and DRUJ. In addition, it was noted that shortening leads to increased pressure within the DRUJ [1, 7, 8].

**Fig. 1:**
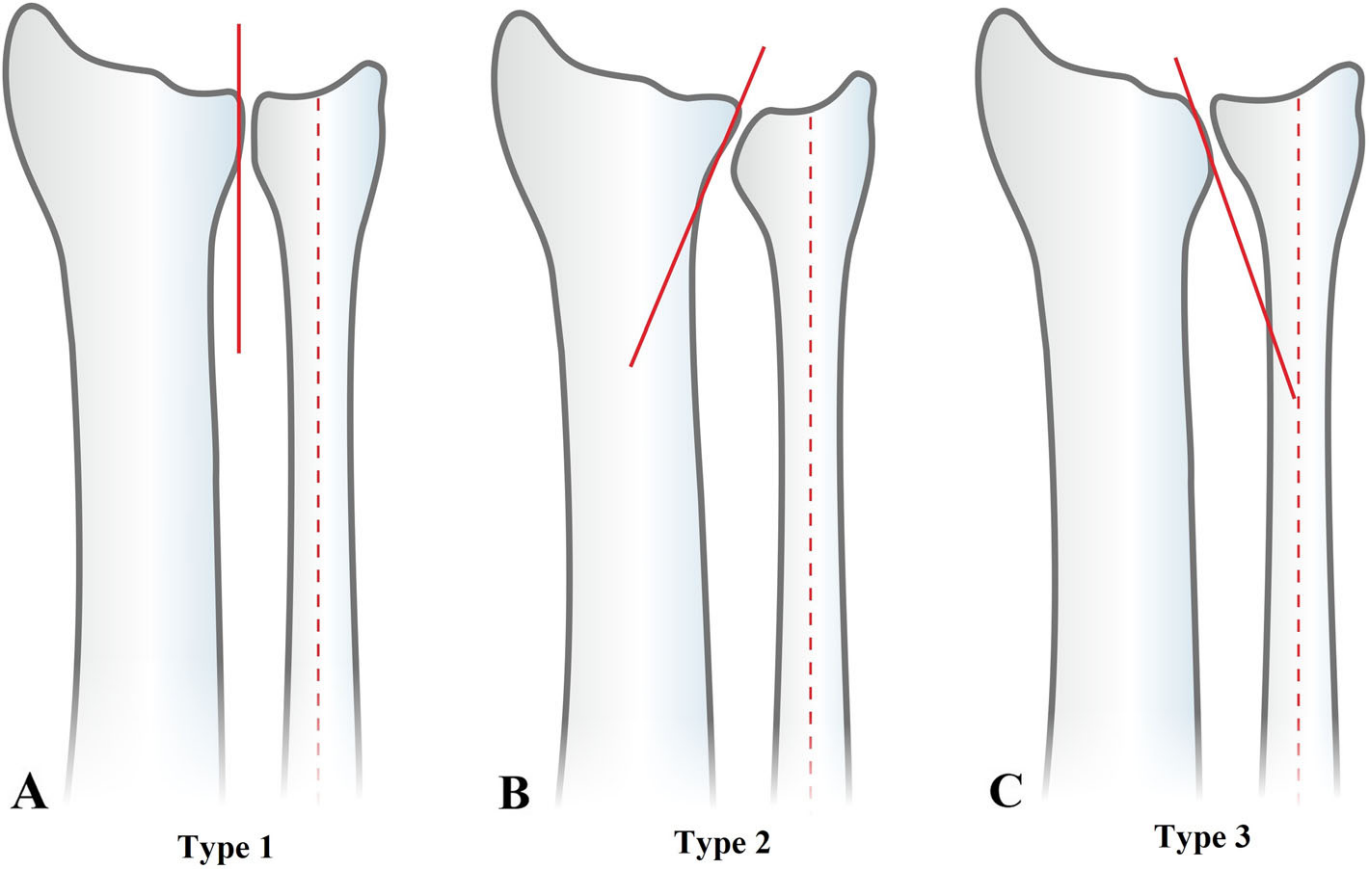
DRUJ Tolat types. **a**. Type 1, parallel: the sigmoid notch is parallel to the long axis of the ulna within ±10°; **b**. Type 2, oblique: the sigmoid notch has an oblique angle facing proximally > 10°; **c**. Type 3, reverse oblique: the sigmoid notch has a reverse oblique angle facing distally > 10

The relationship between the inclination of the sigmoid notch and the ulnar shaft was classified into three Tolat types: type 1, parallel; type 2, oblique; type 3, reverse oblique (Fig. 1) [9]. There is a concern that if USO is carried out on a wrist with a type 3 reverse oblique pattern sigmoid notch, it could increase the contact pressure at the proximal aspect of the inverted sigmoid notch. Thus, it is suspected that accelerated degenerative changes occur due to the contact of unmatched joint surfaces [10, 11].

The percentage of type 3 reverse oblique sigmoid notches was reported to be about 4 to 19% of enrolled non-injured wrists [9, 12, 13]. Hollevoet et al. analysed 248 wrists (248 patients) and found that 81% of the reverse oblique sigmoid notches appeared to have positive ulnar variance, and 19% had neutral ulnar variance [12]. Ulnar impaction syndrome is usually associated with positive ulnar variance, and USO is an effective treatment [5, 14, 15]. As mentioned above, it would not be difficult to encounter a patient with ulnar impaction syndrome combined with a reverse oblique sigmoid notch. However, a literature review found that reports on management of OA of the DRUJ following USO for ulnar impaction syndrome were scarce.

Therefore, our hypothesis was that performing USO in patients with a reverse oblique sigmoid notch would not be harmful to the DRUJ. Our objectives were to evaluate the radiographic changes and functional outcomes after USO in patients with ulnar impaction and a reverse oblique sigmoid notch inclination.

## Methods

This retrospective case series was approved by the ethics committee of our institution. The study adhered to the STROBE guidelines for observational studies [16].

We reviewed patients who had reverse oblique inclination of the sigmoid notch (Tolat type 3) and who underwent USO for ulnar impaction syndrome between 2002 and 2013. The diagnosis of ulnar impaction syndrome relies mainly on clinical examination including the ulnocarpal stress test and the fovea test [17]. We included patients with well-documented clinical records and radiographic evaluations, and with a minimum follow-up period of 3 years. Patients with a neurologic deficit involving the same upper extremity, immunological disease, or renal failure under dialysis were excluded because there could be a misleading functional result related to these diseases.

All surgeries were performed in our hospital by three senior hand surgeons classified as level III (experienced specialist) according to Tang’s grading [18]. A total of 250 USO procedures were performed by these surgeons during the study period. The surgical incision was made between the flexor carpi ulnaris and the extensor carpi ulnaris muscles, beginning at the distal third of the forearm and extending proximally. Shortening osteotomy was performed at the distal third of the ulnar shaft, and a limited-contact dynamic compression plate (LC-DCP) was used for fixation. The indication for USO among the enrolled patients was static or dynamic positive ulnar variance. The goal of the USO was to reach a mild negative ulnar variance (0–1 mm) on the posteroanterior view.

### Radiographic evaluations

During postoperative follow-up, radiographic examinations were performed at the first month and once every month thereafter until 3 months after the junction had healed. Check-ups were then arranged with annual follow-ups. The posteroanterior radiographs of the wrist were taken with the shoulder in 90° abduction, the elbow in 90° flexion, and the wrist in the neutral rotation position. The inclination angle of the sigmoid notch was measured between the long axis of the ulna (> 10 cm of the ulnar shaft presented on the radiograph) and the line of the bony sigmoid notch on the posteroanterior radiographs [10]. The measurements were performed by two independent observers blinded to the results. Intra-observer reliability was evaluated by asking the observer to repeat the assessment after a period of 1 month to minimise recall. The measurements were taken three times. Six data items were available after the two observers had each taken three measurements. We took the median of two numbers as our final data.

### Functional evaluations

The shortened disabilities of the arm, shoulder, and hand questionnaire (QuickDASH) and modified Mayo Wrist Score were used for the final evaluations. The QuickDASH ratings are as follows: excellent (< 20 points), good (20–39 points), fair (40–60 points), or poor (> 60 points). The modified Mayo Wrist Score ratings are excellent (90–100 points), good (80–89 points), fair (65–79 points), or poor (< 65 points) [19–22]. The visual analogue scale (VAS) for pain (from 0 [no pain] to 10 [worst pain]) at rest and during activity were also evaluated. Grip strength was evaluated using a Jamar dynamometer (Sammons Preston, Bolingbrook, IL, USA) set to the second position. The forearm pronation and supination angles were measured using the hand-held pencil method [23, 24], and wrist flexion and extension were measured by placing the goniometer on the dorsum of the patient’s wrist. The level of activity to which each patient returned was also recorded on the basis of a self-reported questionnaire.

### Statistical evaluations

Data are presented as means and standard deviations (SD) for continuous response variables. We analysed the pre- and postoperative functional results using the Wilcoxon signed-rank test. The Mann-Whitney U test was used for comparisons between patients with OA changes of the DRUJ and those who did not have OA changes. The significance level was set at *p* < 0.05. All statistical analyses were performed using the statistical software SPSS (IBM SPSS Statistics for Windows. Version 24.0. Armonk, NY: IBM Corp, 2016).

## Results

**Fig. 2:**
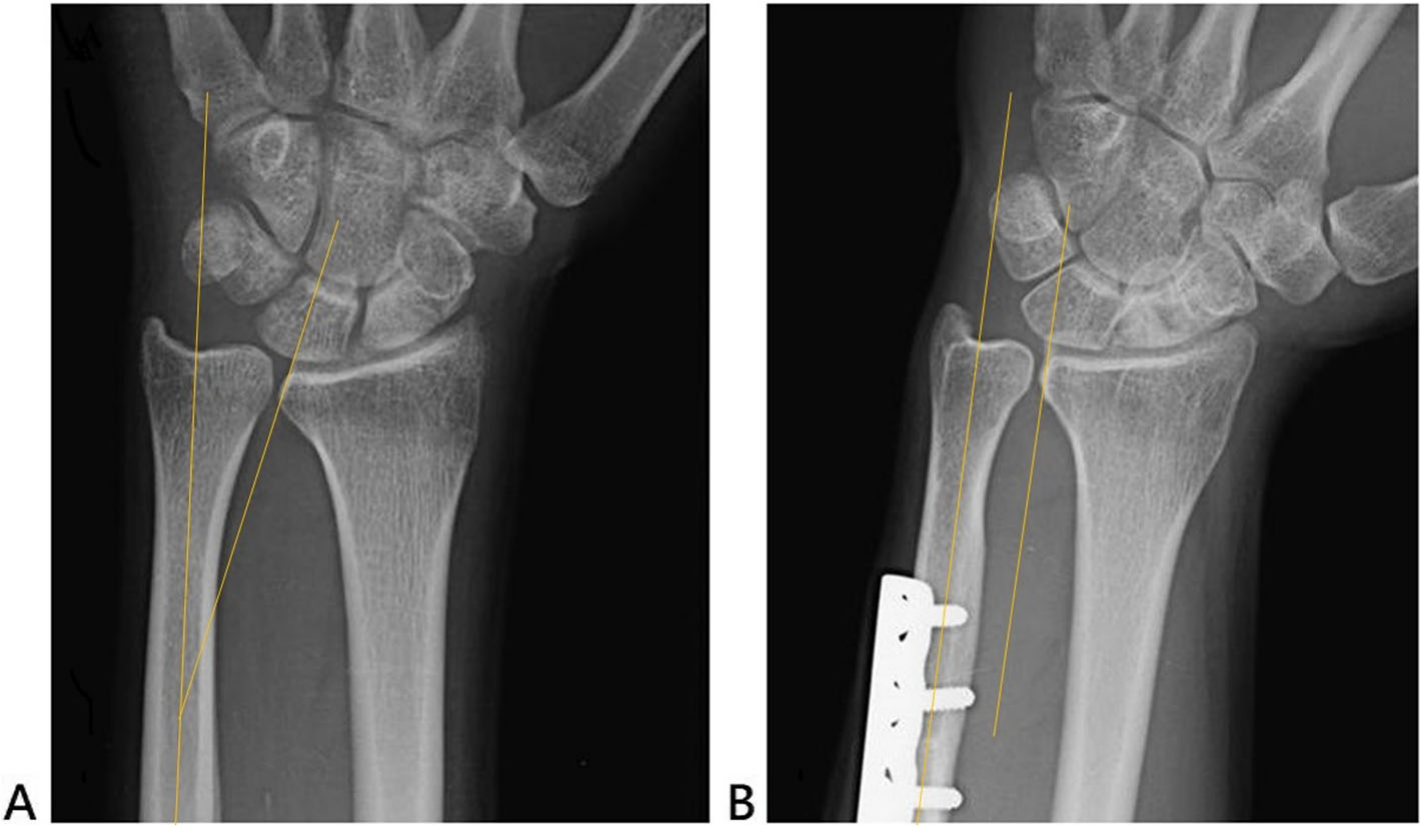
A 27-year-old male patient. **a**. Radiograph of the left wrist before USO showing the reverse oblique sigmoid notch; **b**. Radiograph at the 36-month follow-up after surgery. The bony inclination of the sigmoid notch had become more parallel

Twenty-two patients (22 wrists) with an average age of 49.6 years (range, 25–63 years; SD, 12.1 years) were enrolled. All enrolled patients achieved bone healing at the site of the osteotomy. The mean follow-up period was 93.2 months (range, 36–179 months; SD, 38.2 months). The mean preoperative ulnar variance was + 4.1 mm (range, + 3 − + 6; SD, 1.0 mm), and the mean shortened length was 3.8 mm (range, 3–6; SD, 0.8 mm). There was no obvious DRUJ laxity noted pre- and postoperatively. We found changes in the sigmoid inclination angle, from an average of reverse oblique 14.9^o^ (range, 11°–23°; SD, 3.4°) preoperatively to a more parallel 5.1° (range, 0°–11°; SD 3.2°) at the final follow-up (Fig. 2). The functional results are shown in Table 1. The mean wrist range of motion, VAS for pain at rest and during activity, and grip strength were significantly better at the final follow-ups than at the preoperative evaluations. The mean QuickDASH and the modified Mayo Wrist Score were excellent in the final follow-ups, but we did not have the preoperative data for comparison. All patients returned to their full level of work and activities with no or mild pain, and no wrist braces were needed.

**Table 1:** Functional evaluations before surgery and at final follow-up

Evaluations	Pre-operation Mean ± SD (Range)	Final follow-up Mean ± SD (Range)	*p*-value
Flexion (^o^)	67.5 ± 6.5 (55–80)	74.6 ± 8.6 (50–90)	0.001
Extension (^o^)	69.3 ± 5.0 (60–80)	76.8 ± 6.5 (65–90)	0.001
Pronation (^o^)	73.0 ± 6.3 (60–80)	78.4 ± 3.2 (70–80)	< 0.001
Supination (^o^)	75.5 ± 4.1 (70–80)	82.5 ± 5.5 (70–90)	< 0.001
VAS at rest	0.8 ± 0.7 (0–2)	0.2 ± 0.4 (0–1)	0.002
VAS during activity	6.0 ± 0.9 (5–8)	1.3 ± 0.9 (0–3)	< 0.001
QuickDASH	NA	15.1 ± 8.8 (2.3–34.1)	–
Modified Mayo Wrist Score	NA	91.6 ± 6.4 (70–100)	–
Grip strength (Kg)	17.9 ± 6.7 (10–33)	28.2 ± 7.7 (19–50)	< 0.001

Abbreviations: *VAS* visual analogue scale, *SD* standard deviation, *NA* not available

*p*-value using the Wilcoxon signed-rank test

Seven wrists (31.8%) had OA changes in the DRUJ. Compared to the patients without obvious OA changes, these seven patients had similar functional outcomes, except for the supination angle (OA changes: 78.6 ± 5.6; without OA: 84.3 ± 4.6; *p* = 0.047) and grip strength (OA changes: 23.9 ± 2.6; without OA: 30.2 ± 8.5; *p* = 0.032) (Table 2). The mean QuickDASH and the modified Mayo Wrist Score were excellent in both groups (with or without OA changes).

**Table 2:** Functional evaluations at final follow-up, comparing the patients with and without osteoarthritic change

Evaluations	Patients (*n* = 7) with DRUJ osteoarthritic change Mean ± SD (Range)	Patients (*n* = 15) without DRUJ osteoarthritic change Mean ± SD (Range)	*p*-value
Age at operation (years)	54.1 ± 8.2 (37–61)	47.5 ± 13.2 (25–63)	0.237
Follow-up period (months)	83.7 ± 32.7 (36–134)	97.7 ± 40.8 (62–179)	0.680
Shortening length (mm)	4.0 ± 0.8 (3–5)	3.7 ± 0.8 (3–6)	0.407
Inclination angle change (^o^)	8.9 ± 1.5 (6–10)	10.1 ± 2.6 (6–15)	0.185
Flexion (^o^)	68.6 ± 11.1 (50–80)	77.3 ± 5.6 (70–90)	0.106
Extension (^o^)	75.0 ± 6.5 (70–85)	77.7 ± 6.5 (65–90)	0.407
Pronation (^o^)	76.4 ± 4.8 (70–80)	79.3 ± 1.8 (75–80)	0.237
Supination (^o^)	78.6 ± 5.6 (70–85)	84.3 ± 4.6 (80–90)	0.047*
VAS in rest	0.3 ± 0.5 (0–1)	0.1 ± 0.4 (0–1)	0.581
VAS during Activity	1.9 ± 0.9 (1–3)	1.0 ± 0.8 (0–2)	0.066
QuickDASH	18.0 ± 12.1 (4.5–34.1)	13.8 ± 6.8 (2.3–27.3)	0.490
Modified Mayo Wrist Score	90.0 ± 9.6 (70–95)	92.3 ± 4.6 (80–100)	0.945
Grip strength (Kg)	23.9 ± 2.6 (19–27)	30.2 ± 8.5 (20–50)	0.032*

Abbreviations: *DRUJ* distal radioulnar joint, *VAS* visual analogue scale, *SD* standard deviation, *NA* not available

*p*-value using Mann-Whitney U-test. *: *p* < 0.05

## Discussion

In this study, we observed that the reverse oblique inclination of the sigmoid notch after USO changed the inclination angle. In our series, the long-term radiographic follow-ups showed OA changes at the DRUJ in one-third of patients, but it seemed not to be correlated with functional results.

Sagerman et al. noted that there is a wide variation between the sigmoid notch inclination and ulnar seat angles. Therefore, articular incongruity could occur following USO in all three Tolat types of sigmoid inclination [10, 11]. Because of articular incongruity, there will be a reduction in the joint contact area, which will lead to an increase in the joint reaction force per unit of contact area. This could then be a contributing factor to the subsequent occurrence of remodelling or OA at the DRUJ. In published reviews, the incidence of DRUJ remodelling or degenerative change after USO varied from 16.7 to 43.3% [3, 5, 21, 22, 25], but these were only radiographic changes without functional impairment (Table 3).

**Table 3:** Reviews of radiographic bony change in the DRUJ and functional impairment after ulnar shortening osteotomies

	Mean Age (years)	Mean shortening distance (mm)	Remodeling / degenerative changes in DRUJ (%)	Mean follow-up duration (months)	Functional impairment (evaluation tools)
Köppel [26]	NA	NA	38.3%	18	NO (Chun and Palmer grading score) [27]
Minami [22]	32 (range, 17–57)	3	28%	35	NO (pain, ROM)
Iwasaki [25]	37.5 (range, 14–67)	2.3	24%	26.3	NO (modified Mayo wrist score) [19]
Baek [3]	45.8 (SD 11.5)	5.3	16.7%	60	NO (Gartland and Werley wrist score) [28]
Tatebe [5]	37 (range, 16–64)	2.4	43.3%	18	NO (Hand20 questionnaire) [29]

Abbreviations: *DRUJ* distal radioulnar joint, *NA* not available, *ROM* range of motion, *SD* standard deviation

As for the asymptomatic bony spur or remodelling noted after USO (Table 3), their follow-up duration after surgery was reported to be 18–60 months. Tatebe et al. reported that most of these bony spurs developed within 18 months postoperatively [5]. In our study, the mean follow-up of the seven patients who developed OA was 83.7 months. This study had a longer follow-up period than those reviewed in Table 3, and our data suggest that our patients have good functional results after a longer period. Wrist function of patients with OA was similar to that of patients without OA, except for the wrist supination angle and grip strength. Therefore, the results of this study concur with previously published papers that the bony changes in the DRUJ after USO might not be indicative of clinical deterioration of wrist function after long-term follow-up.

Ross et al. reported that there is no reverse oblique inclination if cartilage thickness is included in the evaluation [13]. For the sigmoid notch with reverse oblique bony inclination, there would be thinner cartilage toward the proximal part of the sigmoid notch, so that the cartilage inclination would no longer be reverse oblique. Deshmukh et al. reported that USO causes DRUJ articular incongruity and reduction in the area of contact of the DRUJ [11]. The proximal part of the sigmoid notch is the contact area after USO in the reverse oblique inclination. Both the increased pressure and thinner sigmoid cartilage at the proximal contact area would cause the DRUJ to develop OA changes. In our study, 31.8% of patients developed OA changes. In addition, we found changes in the sigmoid inclination angle. Without magnetic resonance imaging (MRI), we were unable to confirm whether these bony inclination changes resulted from proximal layer cartilage wear or from a response to the increased force transmitted through the cartilage. However, our results showed that these bony changes did not cause symptoms.

The main limitation of this study is its retrospective nature. The sample size was small, and the results showed a lack of functional differences between those with and without osteoarthritic change. Complete preoperative functional evaluations were unavailable. Because of the retrospective nature of this study, we did not use arthroscopy or other imaging modalities such as MRI or computed tomography that could have provided more information regarding cartilage thickness and changes in bony inclination, which would clarify DRUJ changes [13, 30, 31]. Inter-observer variability could also be a source of bias. It would be interesting to have a longer follow-up to identify if USO influences degeneration of the DRUJ and wrist.

## Conclusions

USO is an effective treatment for ulnar impaction syndrome. For patients with reverse oblique sigmoid inclination after USO, we observed that the inclination angle tends to become parallel and one-third of the patients develop OA changes at the DRUJ, but with good long-term functional outcomes. Based on our findings, reverse oblique sigmoid inclination does not seem to be an absolute contraindication for USO. Further comparative studies are needed to evaluate the impact of USO in patients with different inclination angles.

## Supplementary Information

**Additional file 1.** Checklist of STROBE criteria.

## Data Availability

The datasets used and/or analyzed during the current study are available from the corresponding author on reasonable request.

## Abbreviations

DRUJ: Distal radioulnar joint
MRI: Magnetic resonance imaging
OA: Osteoarthritis
QuickDASH: The shortened disabilities of the arm, shoulder, and hand questionnaire
SD: Standard deviation
TFCC: Triangular fibrocartilage complex
USO: Ulnar shortening osteotomy
VAS: Visual analogue scale
