# Supplementary material for: Long-term radiographic outcomes and functional evaluation of ulnar shortening osteotomy in patients with ulnar impaction syndrome and reverse oblique sigmoid notch: a retrospective case series study
Source: BMC Musculoskelet Disord. 2021 Feb 3;22:136. doi: 10.1186/s12891-021-04029-7 (PMC7860176; doi:10.1186/s12891-021-04029-7)
Supplement: Supplementary file 1 — Additional file 1. Checklist of STROBE criteria. [file 12891_2021_4029_MOESM1_ESM.doc]

STROBE Statement—Checklist of items that should be included in reports of ***cohort studies***

|  | Item No | Recommendation | Present in this paper | Relevant text from manuscript |
| --- | --- | --- | --- | --- |
| **Title and abstract** | 1 | (*a*) Indicate the study’s design with a commonly used term in the title or the abstract | √ | Line 3 |
|  |  | (*b*) Provide in the abstract an informative and balanced summary of what was done and what was found | √ | Lines 34-49 |
| Introduction | | |  |  |
| Background/rationale | 2 | Explain the scientific background and rationale for the investigation being reported | √ | Lines 52-77 |
| Objectives | 3 | State specific objectives, including any prespecified hypotheses | √ | Lines 78-81 |
| Methods | | |  |  |
| Study design | 4 | Present key elements of study design early in the paper | √ | Lines 84-94 |
| Setting | 5 | Describe the setting, locations, and relevant dates, including periods of recruitment, exposure, follow-up, and data collection | √ | Lines 87-97 |
| Participants | 6 | (*a*) Give the eligibility criteria, and the sources and methods of selection of participants. Describe methods of follow-up | √ | Lines 89-94  Lines 106-108 |
|  |  | (*b*) For matched studies, give matching criteria and number of exposed and unexposed | N/A |  |
| Variables | 7 | Clearly define all outcomes, exposures, predictors, potential confounders, and effect modifiers. Give diagnostic criteria, if applicable | √ | Lines 113-118  Lines 120-126 |
| Data sources/ measurement | 8* | For each variable of interest, give sources of data and details of methods of assessment (measurement). Describe comparability of assessment methods if there is more than one group | √ | Lines 124-125, 129 |
| Bias | 9 | Describe any efforts to address potential sources of bias | √ | Lines 113-118 |
| Study size | 10 | Explain how the study size was arrived at | √ | Line 97 |
| Quantitative variables | 11 | Explain how quantitative variables were handled in the analyses. If applicable, describe which groupings were chosen and why | √ | Line 133 |
| Statistical methods | 12 | (*a*) Describe all statistical methods, including those used to control for confounding | √ | Lines 133-136 |
|  |  | (*b*) Describe any methods used to examine subgroups and interactions | √ | Lines 133-137 |
|  |  | (*c*) Explain how missing data were addressed | √ | Lines 90-92 |
|  |  | (*d*) If applicable, explain how loss to follow-up was addressed | N/A |  |
|  |  | (*e*) Describe any sensitivity analyses | N/A |  |
| Results | | |  |  |
| Participants | 13* | (a) Report numbers of individuals at each stage of study—eg numbers potentially eligible, examined for eligibility, confirmed eligible, included in the study, completing follow-up, and analysed | √ | Lines 141-142 |
|  |  | (b) Give reasons for non-participation at each stage | √ | Line 94 |
|  |  | (c) Consider use of a flow diagram | N/A |  |
| Descriptive data | 14* | (a) Give characteristics of study participants (eg demographic, clinical, social) and information on exposures and potential confounders | √ | Lines 141-146 |
|  |  | (b) Indicate number of participants with missing data for each variable of interest | N/A |  |
|  |  | (c) Summarise follow-up time (eg, average and total amount) | √ | Lines 143-144 |
| Outcome data | 15* | Report numbers of outcome events or summary measures over time | √ | Lines 146-155 |
| Main results | 16 | (*a*) Give unadjusted estimates and, if applicable, confounder-adjusted estimates and their precision (eg, 95% confidence interval). Make clear which confounders were adjusted for and why they were included | √ | Lines 146-149 |
|  |  | (*b*) Report category boundaries when continuous variables were categorized | N/A |  |
|  |  | (*c*) If relevant, consider translating estimates of relative risk into absolute risk for a meaningful time period | N/A |  |
| Other analyses | 17 | Report other analyses done—eg analyses of subgroups and interactions, and sensitivity analyses | √ | Lines 156-161 |
| Discussion | | |  |  |
| Key results | 18 | Summarise key results with reference to study objectives | √ | Lines 164-167 |
| Limitations | 19 | Discuss limitations of the study, taking into account sources of potential bias or imprecision. Discuss both direction and magnitude of any potential bias | √ | Lines 202-210 |
| Interpretation | 20 | Give a cautious overall interpretation of results considering objectives, limitations, multiplicity of analyses, results from similar studies, and other relevant evidence | √ | Lines 168-176 |
| Generalisability | 21 | Discuss the generalisability (external validity) of the study results | √ | Lines 213-218 |
| Other information | | |  |  |
| Funding | 22 | Give the source of funding and the role of the funders for the present study and, if applicable, for the original study on which the present article is based | √ | Lines 235-236 |

*Give information separately for exposed and unexposed groups.

**Note:** An Explanation and Elaboration article discusses each checklist item and gives methodological background and published examples of transparent reporting. The STROBE checklist is best used in conjunction with this article (freely available on the Web sites of PLoS Medicine at http://www.plosmedicine.org/, Annals of Internal Medicine at http://www.annals.org/, and Epidemiology at http://www.epidem.com/). Information on the STROBE Initiative is available at http://www.strobe-statement.org.
